# Association of infarct volume with early neurological deterioration in ischemic stroke patients undergoing endovascular treatment

**DOI:** 10.3389/fneur.2026.1755496

**Published:** 2026-04-02

**Authors:** Jianqiang Hu, Jiawei Zhang, Chao Cai, Kefangyuan Zheng, Mingqing Cheng, Xin Miao, Jiarui Bao, Donghua Xian, Yalan Fang, Jin Zhang

**Affiliations:** 1 Department of Neurology, Second Hospital of Shanxi Medical University, Taiyuan, Shanxi, China; 2 Clinical College, Shanxi Medical University, Taiyuan, Shanxi, China; 3 Department of Neurology, First Hospital of Shanxi Medical University, Taiyuan, Shanxi, China

**Keywords:** early neurological deterioration, endovascular treatment, infarct volume, ischemic stroke, prognosis

## Abstract

**Background:**

Early neurological deterioration (END) remains a frequent and serious complication after endovascular treatment (EVT) for ischemic stroke (IS), even in patients with successful recanalization. The role of infarct volume in predicting END, particularly in the EVT population, has not been fully clarified.

**Methods:**

This retrospective study included IS patients who underwent EVT between January 2020 and June 2025. Clinical, laboratory, procedural, and imaging data were collected. Infarct volume was quantified using 3D-Slicer. END was defined as an NIHSS increase ≥4 points within 24 h post-EVT. Logistic regression analyses were used to identify independent predictors, and ROC curves were applied to evaluate the predictive performance of infarct volume.

**Results:**

A total of 682 patients were included, of whom 208 (30.5%) developed END. Multivariate analysis identified systolic blood pressure, atrial fibrillation, triglycerides, the number of EVTs, and infarct volume as independent predictors of END. Infarct volume showed significant predictive value for END (AUC = 0.768), non-hemorrhagic END (AUC = 0.682), and especially hemorrhagic END (AUC = 0.825), with an optimal threshold of 35.5 mL. A nomogram incorporating these independent factors was developed to facilitate individualized risk prediction.

**Conclusion:**

Infarct volume is an independent predictor of END after EVT, with particularly strong predictive value for hemorrhagic END. Incorporating infarct volume into postoperative risk assessment may improve early identification and management of high-risk patients.

## Introduction

Ischemic stroke (IS) remains one of the leading causes of mortality and long-term disability worldwide (1). The advent and widespread implementation of endovascular treatment (EVT) have significantly improved recanalization rates and functional outcomes in patients with large vessel occlusion (LVO) (2, 3). Despite successful reperfusion, however, a considerable proportion of patients experience early neurological deterioration (END) within the first 24 h after EVT. Reported incidence rates range from 10 to 40% (4–6), and END has been consistently associated with increased in-hospital mortality, prolonged hospitalization, and unfavorable long-term functional outcomes (7). Therefore, early identification of patients at high risk of END is of substantial clinical importance.

Previous studies have identified multiple clinical and imaging-related factors associated with END, including advanced age, higher baseline NIHSS score, elevated blood pressure, atrial fibrillation, incomplete reperfusion, and periprocedural complications (8, 9). Nevertheless, reliable biomarkers for early risk stratification remain limited.

Infarct volume represents the extent of irreversible brain tissue injury and reflects the cumulative impact of ischemia before and during reperfusion. Growing evidence suggests that infarct volume is strongly associated with long-term functional outcomes and mortality after EVT (10); However, most existing studies have focused primarily on 90-day functional outcomes rather than early neurological dynamics. Whether infarct volume independently predicts END—particularly in patients who have achieved successful recanalization—remains insufficiently clarified. Moreover, limited data are available regarding its differential predictive value for hemorrhagic and non-hemorrhagic END.

Therefore, the present study aimed to investigate the association between infarct volume and END in patients with acute ischemic stroke undergoing EVT, and to evaluate its predictive performance for overall END and its subtypes. By clarifying the role of infarct volume in early postoperative neurological deterioration, we sought to provide evidence to support imaging-based risk stratification and individualized postoperative management.

## Methods

### Study population

This retrospective study was approved by the Ethics Committee of the First Hospital of Shanxi Medical University. Patients who underwent EVT for AIS between January 2020 and June 2025 were screened.

Inclusion criteria were:

1. EVT performed within 24 h of onset;
2. age ≥18 years;
3. large artery severe stenosis or occlusion confirmed by digital subtraction angiography (DSA).

Exclusion criteria were:

1. pre-stroke mRS >2;
2. posterior circulation large artery occlusion;
3. absence of postoperative head CT;
4. incomplete recanalization (TICI <2b).

Exclusion criteria included patients with incomplete recanalization (TICI <2b), as they were unlikely to experience significant post-reperfusion neurological deterioration.

### Baseline data collection

Collected data included demographic variables (age, sex, height, weight), clinical characteristics (smoking status, admission blood pressure, preoperative NIHSS score, intravenous thrombolysis), medical history (hypertension, history of ischemic stroke, coronary artery disease), and laboratory characteristics (lipid profile, RBC count, neutrophil count, platelets, urea, HbA1c, homocysteine, albumin, fasting glucose). Procedural characteristics were retrieved from operative documentation, including onset-to-puncture time (OPT) and number of EVTs such as aspiration thrombectomy, stent retrieval, balloon angioplasty, stenting, and intra-arterial thrombolysis.

### Imaging analysis

Infarct volume was measured using 3D-Slicer software.^1^ The infarct area on each slice was manually segmented and multiplied by slice thickness to obtain slice volume; these were summed to calculate total infarct volume. CT scans performed 3–7 days post-EVT were prioritized to ensure clear lesion boundaries. When HT occurred, the total infarct volume was first calculated, followed by the calculation of the hemorrhagic area volume. The non-hemorrhagic volume was derived by subtracting the hemorrhagic area volume from the total infarct volume.

The ischemic infarct pattern was categorized into: (1) subcortical regions (whether or not cortical areas are involved); (2) only cortical regions. Subcortical infarction areas included the caudate nucleus, putamen, internal capsule, and thalamus.

All patients underwent CT within 24 h after EVT and repeated CT 3–7 days later to differentiate intracranial hemorrhage from contrast staining.

### Definition of early neurological deterioration

END was defined as an increase of ≥4 points in NIHSS score within 24 h after EVT (11). Hemorrhagic END was diagnosed when intracranial hemorrhage accompanied neurological worsening.

### Statistical analysis

**Figure 1:**
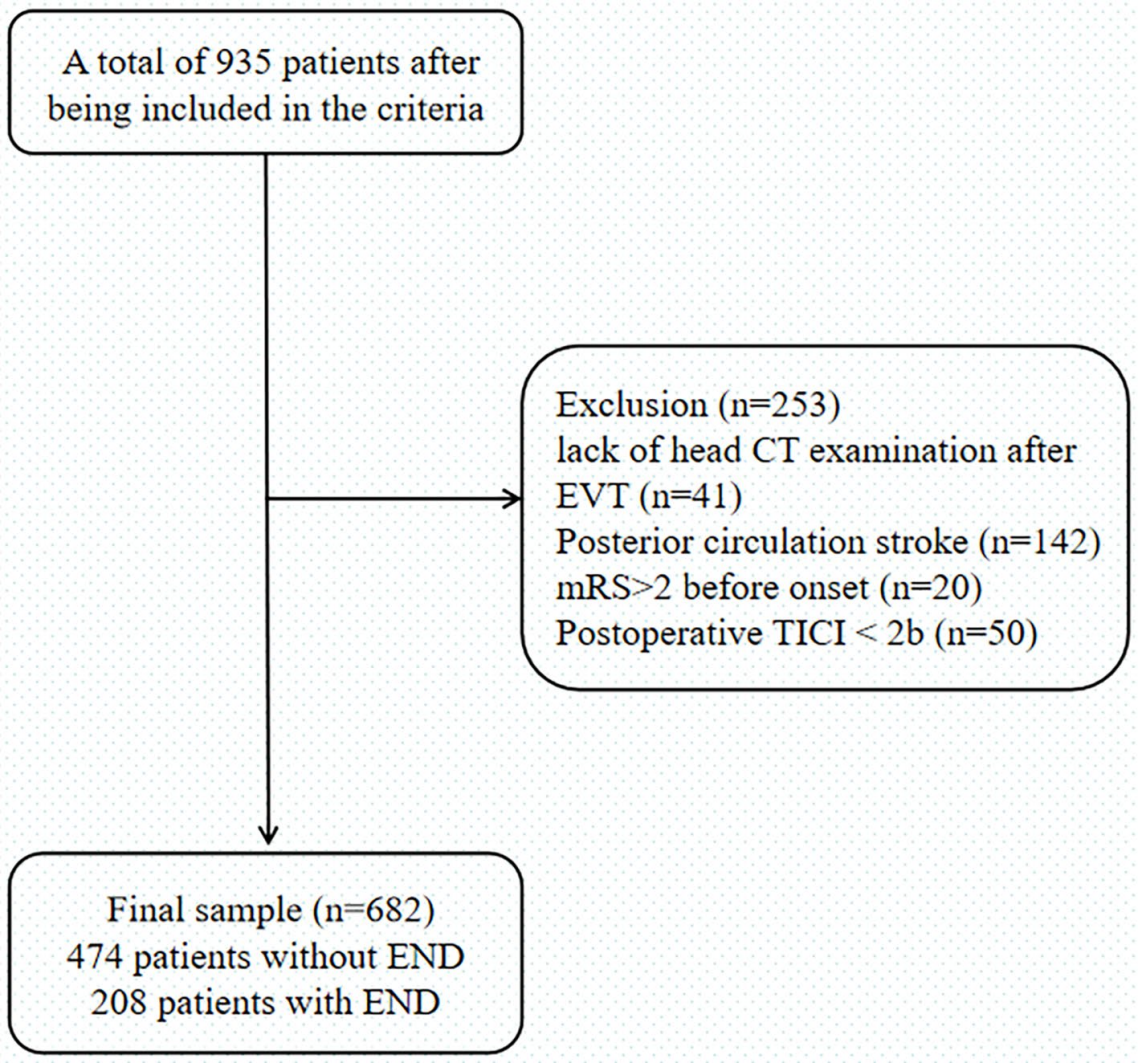
Flow chart of participants’ selection.

Continuous variables were summarized as mean ± standard deviation (SD) or median with interquartile range (IQR), as appropriate, and categorical variables as counts and percentages. Appropriate statistical tests were applied for between-group comparisons according to data type and distribution. For END, non-hemorrhagic END, and hemorrhagic END, univariate and multivariate logistic regression analyses were conducted. Variables with *p* < 0.05 in univariate analyses were included in the multivariate models. Odds ratios (ORs) with 95% confidence intervals (CIs) were calculated to identify independent predictors. The predictive performance of infarct volume for END and its subtypes was evaluated using receiver operating characteristic (ROC) curves. A nomogram incorporating the independent predictors was constructed using the rms package in R to facilitate individualized prediction. Statistical significance was set at a two-sided *p*-value ≤0.05. All statistical analyses were performed using R version 4.1.3 and IBM SPSS Statistics 27 (Figure 1).

## Results

A total of 682 patients met study criteria, including 474 without END and 208 with END.

### Baseline characteristics and risk factors

Baseline comparisons (Table 1) showed that age, systolic blood pressure, baseline NIHSS score, intravenous thrombolysis, medical history (hypertension, atrial fibrillation), laboratory markers (triglycerides, neutrophils, albumin, fasting glucose), and imaging features (infarct volume and location) differed significantly between the END and non-END groups. Characteristics across END subtypes are shown in Supplementary Table S1.

**Table 1:** Baseline characteristics according to the presence of END.

Variables	All patients (*n* = 682)	Without END (*n* = 474)	With END (*n* = 208)	*p*-value
Demographic characteristics				
Age mean ± SD, years	65.66 ± 12.72	65.05 ± 12.88	67.03 ± 12.28	0.062
Male *n* (%)	452 (66.28)	323 (68.14)	129 (62.02)	0.119
Height mean ± SD, cm	167.05 ± 7.66	167.34 ± 7.40	166.40 ± 8.21	0.160
Weight mean ± SD, kg	68.33 ± 12.66	68.32 ± 12.73	68.36 ± 12.53	0.973
Clinical characteristics				
Smoking *n* (%)	302 (44.28)	209 (44.09)	93 (44.71)	0.881
SBP mean ± SD, mmHg	141.20 ± 21.69	139.96 ± 20.36	144.02 ± 24.26	**0.036**
DBP mean ± SD, mmHg	81.68 ± 14.17	81.48 ± 14.19	82.14 ± 14.16	0.572
Preoperative NIHSS median (IQR)	11 (8–15)	11 (7–14)	13 (9–17)	<**0.001**
Intravenous thrombolysis *n* (%)	175 (25.66)	111 (23.42)	64 (30.77)	**0.043**
Medical history				
Hypertension *n* (%)	409 (59.97)	266 (56.12)	143 (68.75)	**0.002**
History of ischemic stroke *n* (%)	153 (22.43)	116 (24.47)	37 (17.19)	0.054
Atrial fibrillation *n* (%)	165 (24.19)	99 (20.88)	66 (31.73)	**0.002**
Coronary heart disease *n* (%)	94 (13.78)	59 (12.45)	35 (16.83)	0.127
Laboratory characteristics				
TC mean ± SD, mmol/L	4.17 ± 1.04	4.18 ± 1.05	4.16 ± 1.03	0.762
TG mean ± SD, mmol/L	1.41 ± 0.85	1.36 ± 0.81	1.54 ± 0.94	**0.009**
LDL-C mean ± SD, mmol/L	2.64 ± 0.78	2.65 ± 0.79	2.60 ± 0.77	0.446
HDL-C mean ± SD, mmol/L	1.09 ± 0.25	1.09 ± 0.24	1.09 ± 0.25	0.946
RBC mean ± SD, 10^12^/L	4.46 ± 0.62	4.46 ± 0.62	4.46 ± 0.60	0.989
Neutrophil mean ± SD, 10^9^/L	6.71 ± 3.01	6.45 ± 2.87	7.31 ± 3.22	<**0.001**
PLT mean ± SD, 10^9^/L	213.57 ± 67.68	214.33 ± 64.82	211.85 ± 73.93	0.659
Urea mean ± SD, mmol/L	5.90 ± 2.85	5.86 ± 2.55	5.97 ± 3.45	0.646
HbA1c mean ± SD, %	6.50 ± 1.52	6.44 ± 1.54	6.61 ± 1.46	0.179
Homocysteine mean ± SD, μmol/L	20.29 ± 15.79	20.42 ± 15.44	19.98 ± 16.60	0.734
Albumin mean ± SD, g/L	39.04 ± 4.33	39.23 ± 4.22	38.51 ± 4.54	**0.036**
Fasting blood sugar mean ± SD, mmol/L	7.32 ± 2.24	7.14 ± 2.14	7.70 ± 2.40	**0.005**
Surgical characteristics				
OPT median (IQR), min	465 (300–720)	465 (300–720)	465 (300–728)	0.765
The number of EVTs median (IQR)	2 (1–3)	2 (1–3)	2 (2–3)	<**0.001**
Imaging characteristics				
Infarct volume median (IQR), mL	25 (9–68)	16 (6–44)	68 (29–176)	<**0.001**
Cortical infarction *n* (%)	199 (29.18)	157 (33.12)	42 (20.19)	<**0.001**
Subcortical infarction *n* (%)	483 (70.82)	317 (66.88)	166 (79.81)	

IQR, interquartile range; SD, standard deviation; END early neurological deterioration; SBP, systolic blood pressure; DBP, diastolic blood pressure; NIHSS, National Institutes of Health Stroke Scale; TG, triglycerides; TC, total cholesterol; LDL-C, low-density lipoprotein cholesterol; HDL-C, high-density lipoprotein cholesterol; RBC, red blood cells; PLT, platelet; HbA1c, hemoglobin A1c; EVT, endovascular treatment; OPT: onset to puncture time. Bold values indicate statistical significance (*P* < 0.05).

### Univariate and multivariate analysis

Univariate analysis identified several variables significantly associated with the occurrence of END, including systolic blood pressure (SBP), preoperative NIHSS score, TG, neutrophil count, infarct location, the number of EVTs and others (all *p* < 0.05). In the multivariate logistic regression model, five factors remained independently associated with END: SBP (OR 1.01, 95% CI 1.00–1.02, *p* = 0.025), atrial fibrillation (OR 2.47, 95% CI 1.55–3.93, <0.001), TG (OR 1.39, 95% CI 1.11–1.74, *p* = 0.004), the number of EVTs (OR 1.37, 95% CI 1.15–1.64, *p* < 0.001), and infarct volume (OR 1.01, 95% CI 1.01–1.02, *p* < 0.001) (Table 2).

**Table 2:** Univariate and multivariate analyses of potential prognostic factors based on END presence.

Variables	Univariate analysis	Multivariate analysis	
	*p*-value	OR (95% CI)	*p*-value
Demographic characteristics			
Age (years)	0.120	—	—
Male	0.063	—	—
Height (cm)	0.143	—	—
Weight (kg)	0.973	—	—
Clinical characteristics			
Smoking	0.881	—	—
SBP (mmHg)	**0.025**	1.01 (1.00–1.02)	**0.025**
DBP (mmHg)	0.571	—	—
Preoperative NIHSS	<**0.001**	0.99 (0.95–1.03)	0.543
Intravenous thrombolysis	**0.044**	1.24 (0.81–1.90)	0.330
Medical history			
Hypertension	**0.002**	1.29 (0.86–1.93)	0.213
History of ischemic stroke	0.055	—	—
Atrial fibrillation	**0.002**	2.47 (1.55–3.93)	<**0.001**
Coronary heart disease	0.128	—	—
Laboratory characteristics			
TC (mmol/L)	0.762	—	—
TG (mmol/L)	**0.010**	1.39 (1.11–1.74)	**0.004**
LDL-C (mmol/L)	0.446	—	—
HDL-C (mmol/L)	0.946	—	—
RBC (10^12^/L)	0.989	—	—
Neutrophil (10^9^/L)	<**0.001**		
PLT (10^9^/L)	0.659	—	—
Urea (mmol/L)	0.646	—	—
HbA1c (%)	0.180	—	—
Homocysteine (μmol/L)	0.734	—	—
Albumin (g/L)	**0.036**	0.97 (0.93–1.02)	0.195
Fasting blood sugar (mmol/L)	**0.004**	1.01 (0.93–1.10)	0.800
Surgical characteristics			
OPT (min)	0.636	—	—
the number of EVTs	<**0.001**	1.37 (1.15–1.64)	<**0.001**
Imaging characteristics			
Infarct volume median (mL)	<**0.001**	1.01 (1.01–1.02)	<**0.001**
Cortical infarction	<**0.001**	1.05 (0.67–1.64)	0.837
Subcortical infarction			

Bold values indicate statistical significance (*P* < 0.05).

For non-hemorrhagic END, TG (OR 1.51, 95% CI 1.18–1.95, *p* = 0.001) and infarct volume (OR 1.01, 95% CI 1.01–1.01, *p* < 0.001) were independent predictors (Table 3).

**Table 3:** Univariate and multivariate analyses for the potential prognostic factors according to the presence of non-hemorrhagic and hemorrhagic END.

Variables	Non-hemorrhagic END (*n* = 83)			Hemorrhagic END (*n* = 125)		
	Univariate analysis	Multivariate analysis		Univariate analysis	Multivariate analysis	
	*p*-value	OR (95% CI)	*p*-value	*p*-value	OR (95% CI)	*p*-value
Demographic characteristics						
Age (years)	0.319	—	—	0.077	—	—
Male	0.580	—	—	0.087	—	—
Height (cm)	0.129	—	—	0.395	—	—
Weight (kg)	0.390	—	—	0.524	—	—
Clinical characteristics						
Smoking	0.935	—	—	0.887	—	—
SBP (mmHg)	0.142	—	—	**0.045**	1.01 (1.00–1.02)	**0.025**
DBP (mmHg)	0.617	—	—	0.669	—	—
Preoperative NIHSS	0.289	—	—	<**0.001**	1.00 (0.95–1.05)	0.982
Intravenous thrombolysis	0.124	—	—	0.109	—	—
Medical history						
Hypertension	**0.007**	1.60 (0.93–2.77)	0.093	**0.039**	1.05 (0.63–1.76)	0.854
History of ischemic stroke	0.941	—	—	**0.010**	0.47 (0.24–0.93)	**0.031**
Atrial fibrillation	0.511	—	—	<**0.001**	3.66 (2.06–6.48)	<**0.001**
Coronary heart disease	0.838	—	—	0.054	—	—
Laboratory characteristics						
TC (mmol/L)	0.833	—	—	0.565	—	—
TG (mmol/L)	**0.003**	1.51 (1.18–1.95)	**0.001**	0.221	—	—
LDL-C (mmol/L)	0.893	—	—	0.252	—	—
HDL-C (mmol/L)	0.959	—	—	0.958	—	—
RBC (10^12^/L)	0.464	—	—	0.580	—	—
Neutrophil (10^9^/L)	0.363	—	—	<**0.001**	1.08 (0.99–1.17)	0.080
PLT (10^9^/L)	0.668	—	—	0.360	—	—
Urea (mmol/L)	0.598	—	—	0.310	—	—
HbA1c (%)	0.333	—	—	0.283	—	—
Homocysteine (μmol/L)	0.467	—	—	0.916	—	—
Albumin (g/L)	0.069	—	—	0.138	—	—
Fasting blood sugar (mmol/L)	**0.009**	1.06 (0.95–1.18)	0.300	**0.042**	1.01 (0.91–1.13)	0.806
Surgical characteristics						
OPT (min)	0.996	—	—	0.510	—	—
The number of EVTs	0.152	—	—	<**0.001**	1.67 (1.34–2.08)	<**0.001**
Imaging characteristics						
Infarct volume median (mL)	<**0.001**	1.01 (1.01–1.01)	<**0.001**	<**0.001**	1.02 (1.01–1.02)	<**0.001**
Cortical infarction	0.332	—	—	<**0.001**	1.21 (0.66–2.22)	0.531
Subcortical infarction						

Bold values indicate statistical significance (*P* < 0.05).

For hemorrhagic END, the multivariate analysis identified SBP (OR 1.01, 95% CI 1.00–1.02, *p* = 0.025), history of ischemic stroke (OR 0.47, 95% CI 0.24–0.93, *p* = 0.031), atrial fibrillation (OR 3.66, 95% CI 2.06–6.48, p < 0.001), and infarct volume (OR 1.02, 95% CI 1.01–1.02, *p* < 0.001) as independent predictors (Table 3).

### Predictive performance of infarct volume

**Figure 2:**
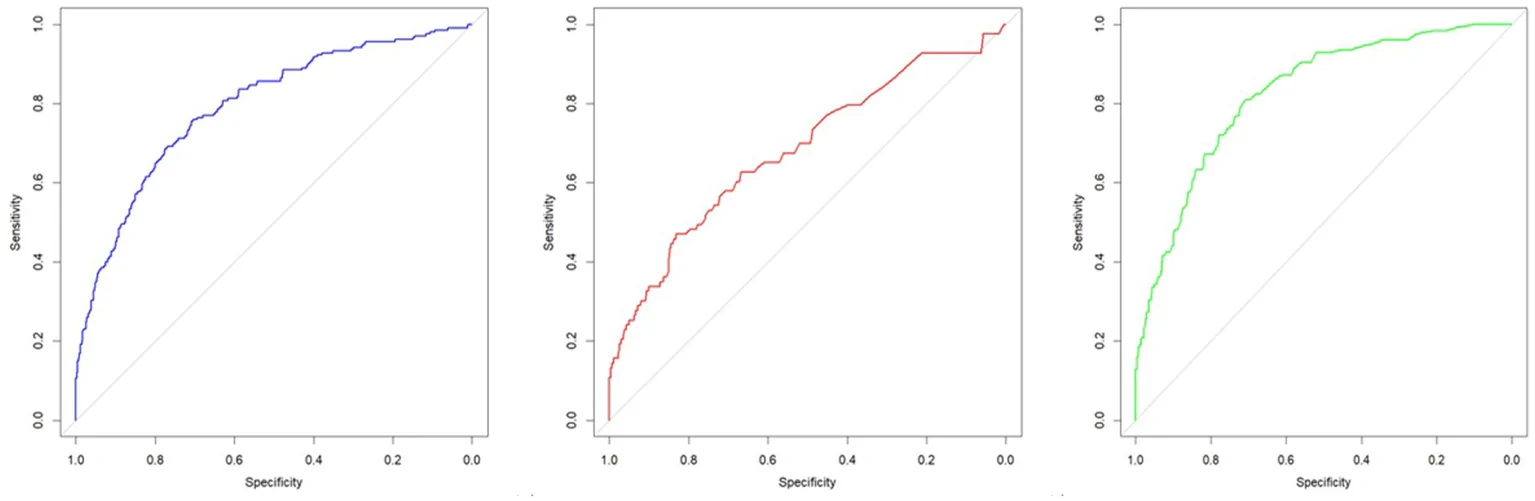
ROC analysis of infarct volume in predicting END, non-hemorrhagic END, and hemorrhagic END.

The predictive performance of infarct volume for END, non-hemorrhagic END, and hemorrhagic END was evaluated using ROC curves (Figure 2). As shown in Table 4, infarct volume showed significant predictive ability for END and its subtypes. Specifically, infarct volume showed good performance in predicting overall END (AUC = 0.768) and hemorrhagic END (AUC = 0.825). Its predictive value was particularly strong for hemorrhagic END, with an optimal threshold of 35.5 mL, a Youden’s index of 0.517, a sensitivity of 0.808, and a specificity of 0.709. In contrast, the ROC analysis showed that infarct volume demonstrated moderate sensitivity (0.831) but low specificity (0.470) for predicting non-hemorrhagic END at a threshold of 58.5 mL. The low specificity indicates a high false-positive rate, suggesting that this threshold may not be clinically useful for reliably identifying patients at risk for non-hemorrhagic END.

**Table 4:** Prediction of END and its subtypes based on infarct volume.

Outcome	AUC	Best threshold (mL)	Youden’s Index	Specificity	Sensitivity
END	0.768	35.5	0.425	0.709	0.716
Non-hemorrhagic END	0.682	58.5	0.301	0.470	0.831
Hemorrhagic END	0.825	35.5	0.517	0.808	0.709

### Nomogram development

**Figure 3:**
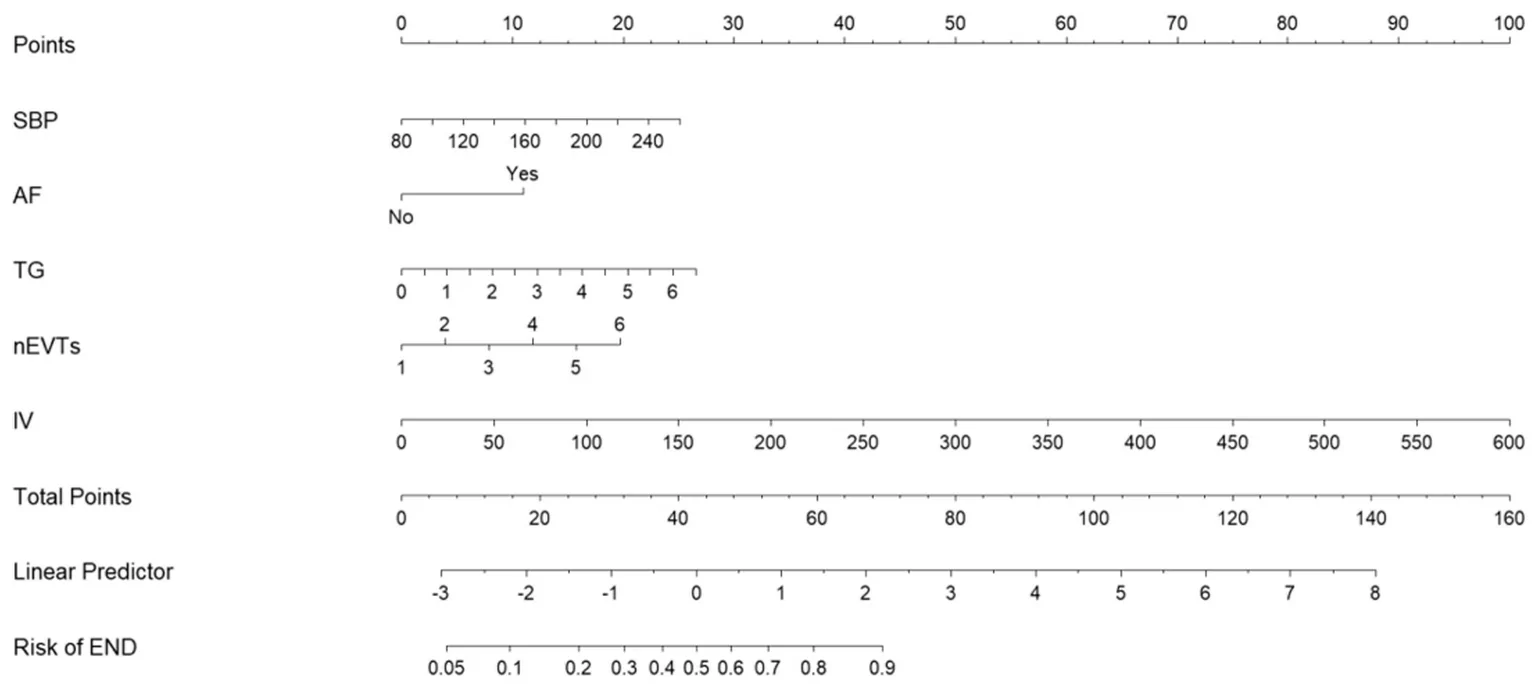
Establishment of a nomogram to predict the incidence of HT.

A nomogram incorporating SBP, atrial fibrillation, triglycerides, infarct volume, and number of EVT maneuvers was constructed to predict END risk (Figure 3).

## Discussion

This study explored the association between infarct volume and END after EVT in IS patients. Our findings demonstrate that infarct volume is an independent predictor of END, with particularly strong discriminative ability for hemorrhagic END.

### Potential mechanisms linking infarct volume and END

The increase in infarct volume is closely associated with the occurrence of END, and this relationship may be driven by multiple pathophysiological mechanisms. First, a larger infarct volume reflects more extensive damage to neural cells and neuronal networks. Massive infarction results in widespread neuronal death, disrupting critical neural transmission pathways and making patients more susceptible to progressive neurological deterioration after the procedure (12, 13).

Second, an enlarged infarct volume is typically accompanied by pronounced cerebral edema. The progressive worsening of edema can lead to elevated intracranial pressure, displacement of local brain tissue, and even midline shift, all of which may rapidly trigger neurological worsening in the early postoperative period (14–16). In patients with large infarcts, edema tends to progress more rapidly after reperfusion, which partly explains the positive association between infarct volume and the incidence of END.

In addition, the microvascular architecture within large infarcted regions is more vulnerable to disruption, increasing the risk of hemorrhagic transformation (HT). HT not only exacerbates cerebral edema but may also directly compress functionally critical neural tissue, thereby producing more profound neurological deterioration (17, 18). This is consistent with our observation that infarct volume demonstrated a relatively strong predictive ability for hemorrhagic END.

Furthermore, when infarct volume reaches a substantial extent, compensatory mechanisms for intracranial pressure regulation may become significantly impaired, leading to instability in cerebral perfusion (19, 20). Meanwhile, our findings suggest that involvement of deep subcortical structures may impose greater functional impairment, further increasing the risk of END.

### Existing research linking infarct volume to functional outcomes and END

Infarct volume has long been recognized as an important imaging marker of irreversible brain tissue damage in acute ischemic stroke and a key determinant of long-term functional outcomes. Prior studies have consistently shown that smaller follow-up infarct volumes (FIV) are associated with better 90-day prognoses. Large pooled analyses, such as those by Boers et al. (21), have identified FIV as an independent predictor of functional recovery after EVT. Similar findings have been reaffirmed in subsequent cohorts, supporting a dose–response relationship between infarct size and outcome, particularly in patients with moderate-to-large infarcts (22, 23). Collectively, these data underscore the clinical relevance of infarct volume as a prognostic biomarker.

However, while the association between infarct volume and long-term functional outcomes has been extensively investigated, evidence regarding its relationship with END remains limited. Existing studies vary markedly in imaging modality, measurement timing, and patient selection, making the current evidence base heterogeneous. Some early work using diffusion-weighted imaging (DWI) within 6 h of onset has suggested that ultra-early lesion volume is an independent predictor of END (24). Other studies have proposed that lesion morphology—such as the “island sign” in perforator artery territory infarction—may supplement or even outperform absolute volume in predicting END, indicating that structural complexity may better reflect tissue vulnerability (25). In addition, recent MRI-based perfusion studies have shown that hypoperfusion burden (e.g., elevated hypoperfusion volume ratio on ASL) is also closely associated with END, further emphasizing that infarct volume alone may not fully capture the dynamic risk of early deterioration (26).

### Clinical implications

This study highlights the importance of infarct volume in predicting early neurological deterioration (END), particularly in the context of hemorrhagic END. However, we also found that the threshold for non-hemorrhagic END (58.5 mL) demonstrated low specificity (0.470), suggesting that it may lead to a high false-positive rate. As a result, using this threshold in clinical practice could result in many patients being incorrectly flagged as high-risk, potentially leading to unnecessary interventions or overmonitoring. Therefore, while infarct volume may serve as a useful predictor, further refinement of this threshold is needed to enhance its clinical applicability.

### Limitations

This study has several limitations. First, infarct volume was measured primarily through manual segmentation, introducing potential subjective bias, and the limited spatial resolution of CT may underestimate small infarcts. Although MRI was used when CT findings were unclear, incomplete MRI availability may still affect measurement accuracy.

Second, previous studies have indicated that patients with incomplete reperfusion are more likely to experience infarct volume progression. Even with successful reperfusion, a small proportion of patients may still experience infarct expansion postoperatively. Although this study excluded cases with incomplete reperfusion (TICI <2b), it is not possible to entirely rule out the possibility of infarct volume progression after partial reperfusion, which could interfere with the final infarct volume assessment (27). Although patients with incomplete recanalization (TICI <2b) were excluded, the possibility of infarct progression after reperfusion cannot be entirely ruled out, which may influence the final infarct volume assessed in this study.

Finally, as a single-center retrospective study, inherent selection and information biases may limit generalizability; multicenter prospective studies are needed to validate these findings.

## Conclusion

In this study of AIS patients undergoing EVT, infarct volume emerged as an independent and clinically meaningful predictor of END, particularly hemorrhagic END. Future multicenter prospective studies are needed to validate these results and further refine imaging-based prediction models for END.

## Data Availability

The original contributions presented in the study are included in the article/Supplementary material, further inquiries can be directed to the corresponding authors.

## Footnotes

1
https://download.slicer.org/
