# Correction: Association of infarct volume with early neurological deterioration in ischemic stroke patients undergoing endovascular treatment

**DOI:** 10.3389/fneur.2026.1978215

**Published:** 2026-09-08

**Authors:** Jianqiang Hu, Jiawei Zhang, Chao Cai, Kefangyuan Zheng, Mingqing Cheng, Xin Miao, Jiarui Bao, Donghua Xian, Yalan Fang, Jin Zhang

**Affiliations:** 1 Department of Neurology, Second Hospital of Shanxi Medical University, Taiyuan, Shanxi, China; 2 Clinical College, Shanxi Medical University, Taiyuan, Shanxi, China; 3 Department of Neurology, First Hospital of Shanxi Medical University, Taiyuan, Shanxi, China

**Keywords:** early neurological deterioration, endovascular treatment, infarct volume, ischemic stroke, prognosis

In the published article, the affiliation Department of Neurology, Second Hospital of Shanxi Medical University was missing for Author Jianqiang Hu.

This affiliation has now been added for author(s) Jianqiang Hu, who should be affiliated with Department of Neurology, Second Hospital of Shanxi Medical University, Taiyuan, Shanxi, China.

The original version of this article has been updated.

