# Correction: Association of infarct volume with early neurological deterioration in ischemic stroke patients undergoing endovascular treatment

**DOI:** 10.3389/fneur.2026.2008304

**Published:** 2026-10-06

**Authors:** Jianqiang Hu, Jiawei Zhang, Chao Cai, Kefangyuan Zheng, Mingqing Cheng, Xin Miao, Jiarui Bao, Donghua Xian, Yalan Fang, Jin Zhang

**Affiliations:** 1 Department of Neurology, Second Hospital of Shanxi Medical University, Taiyuan, Shanxi, China; 2 Clinical College, Shanxi Medical University, Taiyuan, Shanxi, China; 3 Department of Neurology, First Hospital of Shanxi Medical University, Taiyuan, Shanxi, China

**Keywords:** early neurological deterioration, endovascular treatment, infarct volume, ischemic stroke, prognosis

The affiliation for author Jianqiang Hu was incorrectly listed in the published article. The correct and only affiliation for the first author should be: Department of Neurology, Second Hospital of Shanxi Medical University, Taiyuan, Shanxi, China. The affiliation “Clinical College, Shanxi Medical University” should be removed. All other authors and their affiliations remain unchanged.

The original version of this article has been updated.

