# Supplementary material for: Association of infarct volume with early neurological deterioration in ischemic stroke patients undergoing endovascular treatment
Source: Front Neurol. 2026 Apr 2;17:1755496. doi: 10.3389/fneur.2026.1755496 (PMC13083025; doi:10.3389/fneur.2026.1755496)
Supplement: Supplementary file 1 [file Table_1.docx]

**Table S1.** Baseline Characteristics according to the presence of non-hemorrhagic and hemorrhagic END

|  | Without END (n=474) | With non-hemorrhagic END (n=83) | P-value | With hemorrhagic END (n=125) | P-value |
| --- | --- | --- | --- | --- | --- |
| Demographic characteristics |  |  | |  | |
| Age mean ± SD, years | 65.05 ± 12.88 | 66.58 ± 12.62 | 0.319 | 67.33 ± 12.09 | 0.076 |
| Male n (%) | 323 (68.14) | 54 (65.06) | 0.580 | 75 (60.00) | 0.086 |
| Height mean ± SD, cm | 167.34 ± 7.40 | 165.96 ± 8.64 | 0.175 | 166.70 ± 7.94 | 0.396 |
| Weight mean ± SD, Kg | 68.32 ± 12.73 | 69.63 ± 12.94 | 0.397 | 67.51 ± 12.24 | 0.525 |
| Clinical characteristics |  |  | |  | |
| Smoking n (%) | 209 (44.09) | 37 (44.58) | 0.935 | 56 (44.80) | 0.887 |
| SBP mean ± SD, mmHg | 139.96 ± 20.36 | 143.65 ± 24.58 | 0.199 | 144.26 ± 24.14 | 0.069 |
| DBP mean ± SD, mmHg | 81.48 ± 14.19 | 82.33 ± 14.85 | 0.618 | 82.02 ± 13.74 | 0.700 |
| Preoperative NIHSS median (IQR) | 11 (7~14) | 12 (7~16) | 0.242 | 13 (10~17) | **<0.001** |
| Intravenous Thrombolysis n (%) | 111 (23.42) | 26 (31.32) | 0.123 | 38 (30.40) | 0.108 |
| Medical History |  |  | |  | |
| Hypertension n (%) | 266 (56.12) | 60 (72.29) | **0.006** | 83 (66.40) | 0.038 |
| History of Ischemic Stroke n (%) | 116 (24.47) | 20 (20.10) | 0.941 | 17 (13.60) | **0.011** |
| Atrial Fibrillation n (%) | 99 (20.88) | 20 (20.10) | 0.561 | 46 (36.80) | **<0.001** |
| Coronary Heart Disease n (%) | 59 (12.45) | 11 (13.25) | 0.858 | 24 (19.20) | 0.052 |
| Laboratory Characteristics |  |  | |  | |
| TC mean ± SD, mmol/L | 4.18 ± 1.05 | 4.21 ± 0.96 | 0.833 | 4.12 ± 1.08 | 0.565 |
| TG mean ± SD, mmol/L | 1.36 ± 0.81 | 1.67 ± 1.00 | **0.002** | 1.46 ± 0.88 | 0.220 |
| LDL-C mean ± SD, mmol/L | 2.65 ± 0.79 | 2.66 ± 0.74 | 0.893 | 2.56 ± 0.79 | 0.253 |
| HDL-C mean ± SD, mmol/L | 1.09 ± 0.24 | 1.09 ± 0.26 | 0.959 | 1.09 ± 0.24 | 0.958 |
| RBC mean ± SD, 10^12/L | 4.46 ± 0.62 | 4.51 ± 0.58 | 0.465 | 4.42 ± 0.61 | 0.581 |
| Neutrophil mean ± SD, 10^9/L | 6.45 ± 2.87 | 6.76 ± 2.93 | 0.363 | 7.67 ± 3.36 | **<0.001** |
| PLT mean ± SD, 10^9/L | 214.33 ± 64.82 | 217.69 ± 71.60 | 0.669 | 207.97 ± 75.48 | 0.346 |
| Urea mean ± SD, mmol/L | 5.86 ± 2.55 | 5.70 ± 3.04 | 0.598 | 6.16 ± 3.70 | 0.307 |
| HbA1c mean ± SD, % | 6.44 ± 1.54 | 6.62 ± 1.51 | 0.333 | 6.61 ± 1.43 | 0.282 |
| Homocysteine mean ± SD, μmol/L | 20.42 ± 15.44 | 19.05 ± 17.62 | 0.466 | 20.59 ± 15.93 | 0.916 |
| Albumin mean ± SD, g/L | 39.23 ± 4.22 | 38.33 ± 4.74 | 0.068 | 38.63 ± 4.41 | 0.138 |
| Fasting blood sugar mean ± SD, mmol/L | 7.14 ± 2.14 | 7.84 ± 2.56 | **0.021** | 7.60 ± 2.30 | **0.041** |
| Surgical Characteristics |  |  | |  | |
| OPT median (IQR), min | 465 (300~720) | 470 (270~760) | 0.712 | 460 (323~690) | 0.484 |
| the number of EVTs median (IQR) | 2 (1~3) | 2 (1~3) | 0.061 | 3 (2~3) | **<0.001** |
| Imaging Characteristics |  |  | |  | |
| Infarct Volume median (IQR), ml | 16 (6~44) | 46 (14~147) | **<0.001** | 82 (39~189) | **<0.001** |
| Cortical Infarction n (%)  Subcortical infarction n (%) | 157 (33.12) | 23 (27.71) | 0.331 | 19 (15.20) | **<0.001** |
|  | 317 (66.88) | 60 (72.29) |  | 106 (84.80) |  |

**Abbreviations:** IQR, interquartile range; SD, standard deviation; END early neurological deterioration; SBP, systolic blood pressure; DBP, diastolic blood pressure; NIHSS, National Institutes of Health Stroke Scale; TG, Triglycerides; TC, Total Cholesterol; LDL-C, Low-Density Lipoprotein Cholesterol; HDL-C, High-Density Lipoprotein Cholesterol; RBC, Red blood cells; PLT, Platelet; HbA1c, Hemoglobin A1c; EVT, Endovascular Treatment; OPT: Onset to Puncture Time
